# G‐CSF/GM‐CSF‐induced hematopoietic dysregulation in the progression of solid tumors

**DOI:** 10.1002/2211-5463.13445

**Published:** 2022-06-09

**Authors:** Kai He, Xi Liu, Robert D. Hoffman, Rong‐Zhen Shi, Gui‐Yuan Lv, Jian‐Li Gao

**Affiliations:** ^1^ School of Medicine, The First Affiliated Hospital Zhejiang University Hangzhou China; ^2^ School of Basic Medical Sciences Zhejiang Chinese Medical University Hangzhou China; ^3^ Yo San University of Traditional Chinese Medicine Los Angeles CA USA; ^4^ Tangqi Branch of Traditional Chinese Medicine Hospital of Yuhang District Hangzhou China; ^5^ School of Pharmaceutical Sciences Zhejiang Chinese Medical University Hangzhou China

**Keywords:** granulocyte colony‐stimulating factor, granulocyte‐macrophage colony‐stimulating factor, hematopoiesis, metastasis, prognostic, solid tumor

## Abstract

There are two types of abnormal hematopoiesis in solid tumor occurrence and treatment: pathological hematopoiesis, and myelosuppression induced by radiotherapy and chemotherapy. In this review we primarily focus on the abnormal pathological hematopoietic differentiation in cancer induced by tumor‐released granulocyte colony‐stimulating factor (G‐CSF) and granulocyte‐macrophage colony‐stimulating factor (GM‐CSF). As key factors in hematopoietic development, G‐CSF/GM‐CSF are well‐known facilitators of myelopoiesis and mobilization of hematopoietic stem cells (HSCs). In addition, these two cytokines can also promote or inhibit tumors, dependent on tumor type. In multiple cancer types, hematopoiesis is greatly enhanced and abnormal lineage differentiation is induced by these two cytokines. Here, dysregulated hematopoiesis induced by G‐CSF/GM‐CSF in solid tumors and its mechanism are summarized, and the prognostic value of G‐CSF/GM‐CSF‐associated dysregulated hematopoiesis for tumor metastasis is also briefly highlighted.

## Graphical abstract

G‐CSF/GM‐CSF are well‐known facilitators of myelopoiesis and mobilization of hematopoietic stem cells. G‐CSF/GM‐CSF‐induced hematopoietic dysregulation has a critical role in the progression and metastasis of solid tumors, characterized by myeloproliferative phenomena, extramedullary hematopoiesis, and bone marrow stem/progenitor compartment destruction.

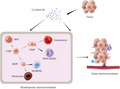

**Abbreviations**
EMH: extramedullary hematopoiesis
G‐CSF: granulocyte colony stimulating factor
GM‐CSF: granulocyte‐macrophage colony stimulating factor
HSC: hematopoietic stem cell
iMC: immature myeloid cell
MDSC: myeloid‐derived suppressor cell
M‐MDSC: monocytic myeloid‐derived suppressor cell
PMN‐MDSC: polymorphonuclear myeloid‐derived suppressor cell
TAM: tumor‐associated macrophage
TAN: tumor‐associated neutrophil
TME: tumor microenvironment

Hematopoiesis is the production of blood cells and immune cells from pluripotent hematopoietic stem cells (HSCs) in hematopoietic organs. This process is precisely controlled by the level of endogenous hematopoietic growth factor and the interplay of transcriptional and epigenetic networks [1]. In general, hematopoiesis is mainly driven by various cytokines such as granulocyte colony‐stimulating factor (G‐CSF), granulocyte‐macrophage colony‐stimulating factor (GM‐CSF), and so on. G‐CSF and GM‐CSF can induce the differentiation of HSCs into granulocyte lineage and monocyte lineage, which is important to build an immune barrier.

In recent years, hematopoietic abnormalities in solid tumors characterized by myeloproliferative phenomena, extramedullary hematopoiesis (EMH), and bone marrow stem/progenitor compartment destruction have attracted more and more attention. It is considered to play an important role in the tumor immunosuppressive milieu, metastasis, and prognosis.

The spleen, one of the major sites of EMH induced by solid tumors, is characterized by an accumulation of various hematopoietic stem/progenitor cell (HSPC) types and is associated with a significant myeloid skew within the Lin^lo/−^Sca‐1^+^c‐Kit^hi^ (LSK) cell population. Evidence suggests that hyperactivation of the CCL2/CCR2 pathway in the spleen of tumor‐bearing mice contributes to the recruitment of LSK HSPCs [2]. Newly suggested evidence reveals that splenic LSK cells derived from Hepa1‐6 hepatoma mouse expressed a high level of GM‐CSF, which induced splenic LSK cells to generate highly suppressive myeloid descendants [2]. These results have indicated that splenic LSK HSPCs readily respond to GM‐CSF signaling and support the suppressive myeloid response under the state of tumor‐bearing.

The liver is one of the primary organs where EMH can take place. A study suggested that an extramedullary niche suitable for HSCs migration and differentiation consist of liver sinusoidal endothelial cells (LSECs) characterized by high expression of stromal‐derived factor (SDF)‐1 [3]. *In vitro*, LSECs have been confirmed to support differentiation, proliferation, and survival of HSCs [3]. Evidence indicated that SDF‐1 mediated activation of the SDF‐1/CXCR4 axis, which is involved in hepatic extramedullary niche formation [4]. Notably, tumor‐derived G‐CSF could enhance the level of hepatocyte SDF‐1, thereby promoting EMH in the liver [4].

Research suggests that the serum levels of cytokines, including G‐CSF, GM‐CSF, and proinflammatory cytokines produced by malignant cells and stromal cells are usually elevated in patients with solid tumor cancers [5]. Subsequently, these cytokines induce hyperhematopoiesis in bone marrow and EMH. This extremely enhanced hematopoiesis causes the production of immature myeloid cells (iMCs) in large numbers. Although a small part of iMCs can differentiate into normal lineage, most iMCs differentiate into tumor‐associated myeloid cells such as tumor‐associated macrophages (TAMs), tumor‐associated neutrophils (TANs), and myeloid‐derived suppressor cells (MDSCs). Therefore, rebuilding hematopoietic balance is a potential new approach for adjuvant therapy in advanced cancer patients [6].

## Different types of tumor‐associated myeloid cells and their phenotypic characteristics

**Fig. 1:**
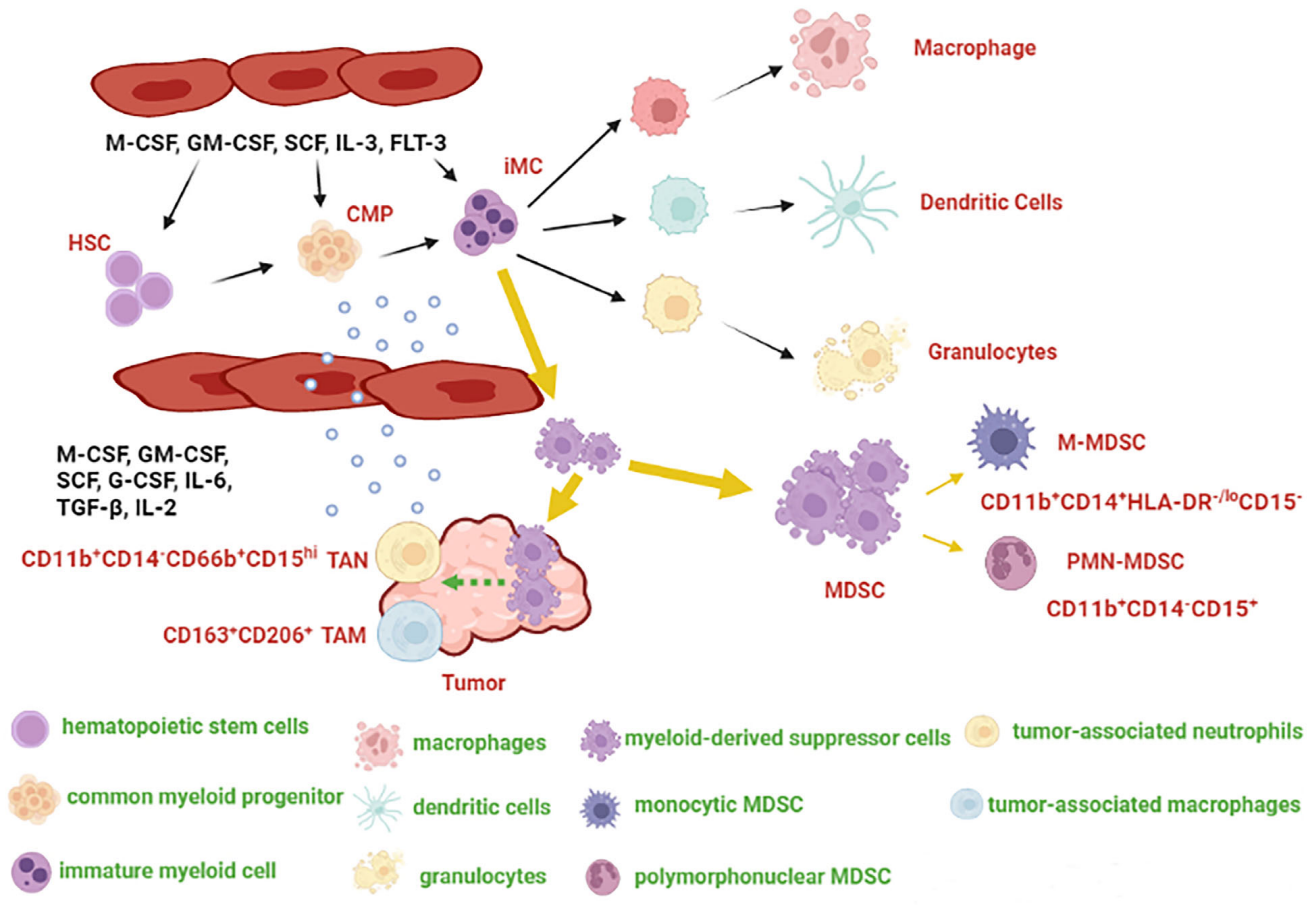
Hematopoietic process in patients with solid tumors. Cancer patients secrete various cytokines in large quantities, including granulocyte colony stimulating factor (G‐CSF), granulocyte and macrophage colony stimulating factor (GM‐CSF), macrophage‐colony stimulating factor (M‐CSF), and so on. Generally speaking, these cytokines promote the differentiation of hematopoietic stem cells (HSCs) into immature myeloid cells (iMCs), and then further differentiate into immune cells such as macrophage, dendritic cells (DCs), and granulocytes. However, the normal differentiation of HSC is blocked in patients with solid tumors, and iMC mainly differentiate into myeloid‐derived suppressor cells (MDSCs) rather than normal immune cells. In addition, MDSC is recruited by tumors and induces the production of tumor‐associated macrophages (TAMs) and tumor‐associated neutrophils (TANs), thereby promoting the formation of the tumor immunosuppressive microenvironment. CMP, common myeloid progenitor; PMN‐MDSC, polymorphonuclear myeloid‐derived suppressor cells; M‐MDSC, monocytic myeloid‐derived suppressor cells; SCF, stem cell factor; TGF‐β, transforming growth factor‐β; IL‐2, interleukin‐2; IL‐3, interleukin‐3; IL‐6, interleukin‐6; FLT‐3, fms‐like tyrosine kinase‐3. [Colour figure can be viewed at wileyonlinelibrary.com]

Tumor‐derived factors regulate the differentiation of HSCs in bone marrow, subsequently contributing to hematopoietic dysregulation in cancer patients. The different hematopoietic processes in patients with malignant tumors are shown in Fig. 1 and the phenotype of tumor‐associated cells is listed in Table 1.

**Table 1:** Phenotypic characteristics of MDSC, TAM, and TAN. MDSC, myeloid‐derived suppressor cell; PMN‐MDSC, polymorphonuclear myeloid‐derived suppressor cells; M‐MDSC, monocytic myeloid‐derived suppressor cells; TAM, tumor‐associated macrophages; TAN, tumor‐associated neutrophils.

Human	Phenotype
PMN‐MDSC	CD14^−^CD11b^+^CD15^+^ [7]
M‐MDSC	CD11b^+^CD14^+^HLA‐DR^−/lo^CD15^−^ [7]
TAM	M1: MHC‐II^+^CD68^+^ [8] M2: CD163^+^CD206^+^ [8]
TAN	CD11b^+^CD14^−^CD66b^+^CD15^hi^ [9]

## Primary features of abnormal hematopoiesis in solid tumor patients

As summarized in (Table 2), tumor‐derived factors can activate bone marrow and promote the differentiation of HSCs, subsequently contributing to abnormal increases of circulating MDSCs. Wu *et al*. [10] discovered that the frequency of circulating granulocyte–monocyte progenitors (GMPs) increased 4–6 times in all tumors examined. The dysfunction of progenitors led to abnormal blood tests in patients with solid tumors, characterized by high white blood cell (WBC) counts, platelets (PLT), eosinophils (EO), and a high neutrophil‐to‐lymphocyte ratio (NLR), as well as the dysregulation of T‐lymphocyte populations. Clinically, tumor‐related hematological abnormalities include tumor‐related anemia, thrombosis, and tumor‐associated immunosuppression.

**Table 2:** Alterations of blood cells in tumor‐bearing hosts. HSC, hematopoietic stem cell; MDSC, myeloid‐derived suppressor cell; WBC, white blood cell; RBC, red blood cell; PLT, platelets; PCT, plateletcrit; EO, eosinophils; BASO, basophils; NURO, neutrophil; NLR, neutrophil‐to‐lymphocyte ratio; PLR, platelet to lymphocyte ratio; NEUT%, percentage of neutrophils; EPO, erythropoietin; TPO, thrombopoietin; PDGF, platelet‐derived growth factor; TGF‐β, transforming growth factor‐β; G‐CSF, granulocyte colony stimulating factor; FLT3L, fms‐like tyrosine kinase‐3 ligand; GM‐CSF, granulocyte and macrophage colony stimulating factor; IL‐3, interleukin‐3; IL‐6, interleukin‐6; M‐CSF, macrophage colony stimulating factor; SCF, stem cell factor; IL‐2, interleukin‐2.

Blood cells	Alteration	Related factors
HSC	Two‐fold increase in seven different types of tumors in human	TGF‐β, G‐CSF, FLT3L, GM‐CSF, IL‐3, IL‐6, TPO, M‐CSF, or a combination of IL‐3/IL‐6/SCF [10]
MDSC	Increase immunosuppression effect	GM‐CSF and IL‐6 [5, 10]
WBC	3‐ to 7‐fold increase in mouse mammary tumors	IL‐2 [11]
RBC	Decreased hematocrit, red blood cells, hemoglobin	IL‐3, IL‐6, TPO, M‐CSF [11]
PLT	PLT and PCT increase in mouse mammary cancer	PDGF and P‐selectin [12]
EO	EO decreases in human breast cancer	LSK [12]
BASO	BASO decreases in mouse mammary tumor	TGF‐β, IL‐3 [12]
NURO	NEUT% decreases in mouse mammary tumor	IL‐3, IL‐6, TPO, M‐CSF [11]
Lymphocyte	NLR and PLR decrease in human breast cancer, the number of Treg increases	EPO [13]

The regulation of the proliferation and differentiation of hematopoietic progenitor cells (HPCs) is generally controlled by hematopoietic growth factors (HGFs), a type of glycoprotein hormone, in the bone marrow. Although not very common, cancers, especially the most rapidly advancing ones, secrete HGFs like G‐CSF and GM‐CSF. For example, tumor cells from acute myeloid leukemia (AML) patients expressed transcripts for HGFs including GM‐CSF and G‐CSF [14]. Furthermore, increased G/GM‐CSF levels are confirmed to be highly correlated with poor prognosis and tumor staging in cancers such as lung cancer, glioma, colorectal cancer, melanoma, breast, and bladder cancers [15]. These factors are not necessarily secreted directly by tumor cells, but may be secreted by tumor‐associated immune cells or stromal cells. The effects of several growth factors, including colony‐stimulating factor (CSFs) and erythropoietin (EPOs), on hematopoiesis have been extensively studied [16]. HGFs and compounds that simulate their actions have complex effects on multiple hematologic cells. Several known HGFs, such as EPO‐α, EPO‐β, G‐CSF, GM‐CSF, interleukin 11 (IL‐11) and thrombopoietin receptor agonists have been used clinically.

G‐CSF and GM‐CSF are well‐known cytokines secreted by immune cells, fibroblasts, and endothelium. In physiological conditions, G‐CSF and GM‐CSF can induce the differentiation of HPCs into different immune cells, including granulocytes, monocyte‐macrophages, T‐cells, and natural killer (NK) cells in the bone marrow. Therefore, they are widely used as potent factors to control radiation‐ and chemotherapy‐induced neutropenia in cancer therapies [17, 18, 19]. However, the potential effect of G‐CSF and GM‐CSF in promoting tumor growth due to their cytokine‐mediated immune suppression and angiogenesis has also been observed [15]. Analysis of clinical data shows that patients with G‐CSF‐ or GM‐CSF‐positive tumors are more likely to experience tumor metastases. In addition, G‐CSF, GM‐CSF and their receptors were expressed to a varying degree in human meningiomas but were not expressed in normal tissue [20]. Previous studies have indicated that G‐CSF or GM‐CSFs can affect differentiation of bone marrow cells and increase the ratio of tumor‐associated immunosuppressive cells through triggering JAK protein kinases phosphorylation and subsequent activation of STAT3/STAT5 transcription factors [21, 22, 23, 24, 25].

### HSCs

Giles *et al*. [26] found increased production and mobilization of HSCs in cancer patients and animal models. A study suggests that tumors can activate the function of bone marrow and mobilize HSCs. These HSCs contribute to forming an immunosuppressive milieu in distant tissue sites before tumor metastases. Therefore, circulating HSCs can be considered potential clinical indicators of metastatic niche formation and be used to monitor the metastatic process in the early and middle stage of malignant tumors [26]. Furthermore, the circulating HSCs exhibited myeloid bias with a skew toward granulocytic differentiation in patients with solid tumors. Wu *et al*. found a 4–7‐fold increase of circulating GMPs in peripheral blood in patients with seven different types of tumors (*n* = 133), which positively correlated with disease progression. These reports reveal the vital role of circulating HSCs in patients with cancer [10].

### MDSCs

MDSCs represent a heterogeneous population of largely immature myeloid cells whose production is induced by tumor‐derived factors, such as G‐CSF, GM‐CSF, and IL‐6. MDSCs have suppressive properties in T‐cell proliferation, cytotoxic and NK cell activation, and also promote the differentiation and expansion of Foxp3^+^ suppressive regulatory T‐cells (Tregs), which are critically involved in immune tolerance and homeostasis. The production and immunosuppressive effect of MDSCs are well described in many diseases including, but not limited to, obesity, cancer, autoimmune disorders, and infection. Indeed, it has been previously proven that MDSCs can be recruited by tumor‐derived chemotactic factors, enter tumor microenvironments, and differentiate into mature immunosuppressive cells represented by TAMs.

In mice, two major subtypes of MDSCs have been identified, including monocytic MDSCs (M‐MDSCs) (CD11b^+^Ly6G^−^Ly6C^hi^) and polymorphonuclear MDSCs (PMN‐MDSCs) (CD11b^+^Ly6G^+^Ly6C^lo^). In humans, there is another subtype called early‐stage MDSCs (e‐MDSCs), which express the myeloid markers CD33 and CD11b but lack the myeloid lineage markers CD14 and CD15/CD66b [27].

MDSCs inhibit antitumor immune response through various mechanisms. L‐Arginine is important for T lymphocyte proliferation and production of interferon‐gamma (IFN‐γ) by NK cells. MDSCs promote the depletion of L‐arginine by arginase 1 (Arg‐1) hypersecretion, thereby suppressing the antitumor effect of T and NK cells [28, 29]. Indoleamine 2,3‐dioxygenase 1 (IDO‐1) is associated with tryptophan degradation. Accumulation of tryptophan degradation products enhances the differentiation of Tregs and its immunosuppressive effect [30]. Moreover, MDSCs highly express iNOS and NADPH oxidase 2 (Nox2), which induce ROS and RNS production. Reactive oxygen species (ROS) and reactive nitrogen species (RNS) inhibit infiltration and reaction of CD8^+^ in the tumor microenvironment (TME) [31].

TAMs and MDSCs are the most myeloid cell populations in TME [32]. Monocytes are precursors of macrophages, and in tumors the population of monocytic cells consists of classical monocytes and M‐MDSCs [33]. Notably, the functional heterogeneity of macrophages in cancer depends on the nature of their precursors; M‐MDSC‐derived macrophages maintain immune‐suppressive activity. A study has indicated that S100A9 was highly expressed in M‐MDSC‐derived macrophages, which drives the polarization of these macrophages to the M2 subtype. In addition, the hyperactivation of the S100A9/C/EBPβ pathway is correlated with the immunosuppressive effect of M2 macrophages [34]. Recent studies have suggested that hypoxia and RAR‐related orphan receptor C (RORC1) were identified as critical in M2 macrophage generation from M‐MDSCs in tumors [35].

### TANs

Neutrophils play a key role in the defense against infection and activation of both the innate and adaptive immunity. In malignant tumors, TANs have been regarded as an important component of TME [36]. In humans, the surface marker of TANs has been identified as CD11b^+^CD14^−^CD66b^+^CD15^hi^ [9]. In 2009, a delineation between antitumorigenic and protumorigenic neutrophils was suggested, termed N1/N2, respectively [37]. N1 neutrophils display cytotoxicity on tumor cells by generating ROS and activating the ROS/TRPM2 pathway, resulting in a lethal influx of calcium ions into the cell [38]. Furthermore, N1 neutrophils stimulate an adaptive immune response and IFN‐γ generation through presenting antigens to T‐cells [39]. Interestingly, N1 neutrophils inhibit tumor progression by antibody‐mediated phagocytosis [40]. Within the TME, Fridlender *et al*. [37] demonstrated that transforming growth factor‐β (TGF‐β) induces the N2 phenotype, while blocking TGF‐β recruits and activates N1 phenotype. Other studies have shown that interferon‐β (IFN‐β) affects tumor angiogenesis and can bias TAN to the N1 phenotype [41]. However, a deficiency of endogenous IFN‐β may allow neutrophils to express higher levels of C‐X‐C chemokine receptor type 4 (CXCR4), vascular endothelial growth factor (VEGF), and matrix metalloproteinase 9 (MMP9), thus promoting angiogenesis, mobility, and tumor homing. Further, Granot *et al*. [42] have shown that the neutrophils in metastatic regions (distal to the primary tumor) can depress tumor growth directly and secrete immunomodulators.

In contrast, N2 neutrophils accelerate tumor progression through various mechanisms. N2 neutrophils release multiple enzymes, including myeloperoxidase (MPO), neutrophil elastase (NE), neutrophil collagenase (MMP8), and gelatinase B (MMP9). MMPs facilitate extracellular matrix remodeling and angiogenesis [43]. NE and MPO accelerate tumor proliferation and migration by regulating neutrophil extracellular traps (NETs) production [44]. In addition, N2 neutrophils recruit Tregs to TME by CCL17 secretion, thereby inhibiting the function of effector T lymphocytes [45]. Some evidence has indicated that N2 neutrophils could reduce tumor growth by inhibiting the production of IL‐17‐secreting γδ T‐cells [46]. Kumagai *et al*. [47] found that surgical stress recruits peripheral immunosuppressive low‐density neutrophils (LDN) in the early postoperative period, which may support the lodging of circulating tumor cells via NETs formation and inhibit T‐cell‐mediated antitumor response in target organs, which may promote postoperative cancer metastases. Although there are functional differences, no clear surface marker has been identified to distinguish N1 and N2 types.

### TAMs

TAMs represent one of the primary tumor‐infiltrating immune cell types, displaying functional plasticity and adapting to change within the microenvironment [48]. In general, macrophages divide into classical activated M1 macrophages (MHC‐II^+^CD68^+^) and alternatively activated M2 macrophages (CD163^+^CD206^+^) [8]. The former exerts antitumor functions and the latter promotes the occurrence and metastasis of tumor cells. M1 macrophages kill tumor cells by releasing tumor‐killing molecules or antibody‐dependent cell‐mediated cytotoxicity (ADCC) [49]. Contrastingly, M2 macrophages facilitate tumor proliferation via expressing various growth factors such as VEGF, TGF‐β1, epithelial growth factor (EGF), and hepatocyte growth factor [50]. In glioma cells, extracellular adenosine deaminase protein in the cat eye syndrome critical region protein 1 (CECR1) has been confirmed to regulate macrophages maturation [51]. M2 macrophages promote tumor invasion through activating the CECR1/MAPK pathway [51]. Furthermore, M2 macrophages directly accelerate tumor metastasis and angiogenesis by generating various soluble factors and proteases, such as serine protease, cathepsin, and MMPs [52]. Thus, M2 macrophages, the predominant type of TAMs, can not only suppress the proliferation of CD8^+^ T‐cells by producing Arg‐1, iNOS, ROS, and RNS, but also inhibit antitumor immune response by recruiting Tregs [49]. Therefore, tumors reprogramed macrophage metabolism to maintain its M2 subtype.

## The mechanism of G‐CSF/GM‐CSF‐induced hematopoietic dysregulation

The first identified HGFs were called CSFs because they could stimulate growth and clone formation of various bone marrow HSCs *in vitro*. G‐CSF has been widely used to mobilize HSCs in the bloodstream for transplantation for over two decades. It efficiently mobilizes HSCs into the blood by targeting HSCs niche function and bone formation via specific bone marrow macrophages.

GM‐CSF is one of the main tumor‐derived soluble factors that can induce the differentiation of monocytic/granulocytic bone marrow cells into CD11b^−^Gr1^−^ bone marrow progenitor cells, subsequently inducing them to differentiate into MDSCs [53]. Many studies suggest that GM‐CSF plays a major role in the suppression of antitumor immune responses and tumor‐mediated dysregulation of hematopoiesis. For example, patients with stage IV metastatic melanoma injected with heat shock protein peptide complex gp96 (HSPPC‐96), GM‐CSF (75 μg subcutaneously into the vaccine site) once a week for 4 weeks, and then once every 2 weeks, could promote the production of new subsets of myeloid suppressor cells (MSCs) [54]. High doses of GM‐CSF administered in a vaccine formulation could also recruit MSCs and substantially inhibit antitumor immunity responses in melanoma mice [55].

### G‐CSF/GM‐CSF‐induced HSCs proliferation and mobilization

**Fig. 2:**
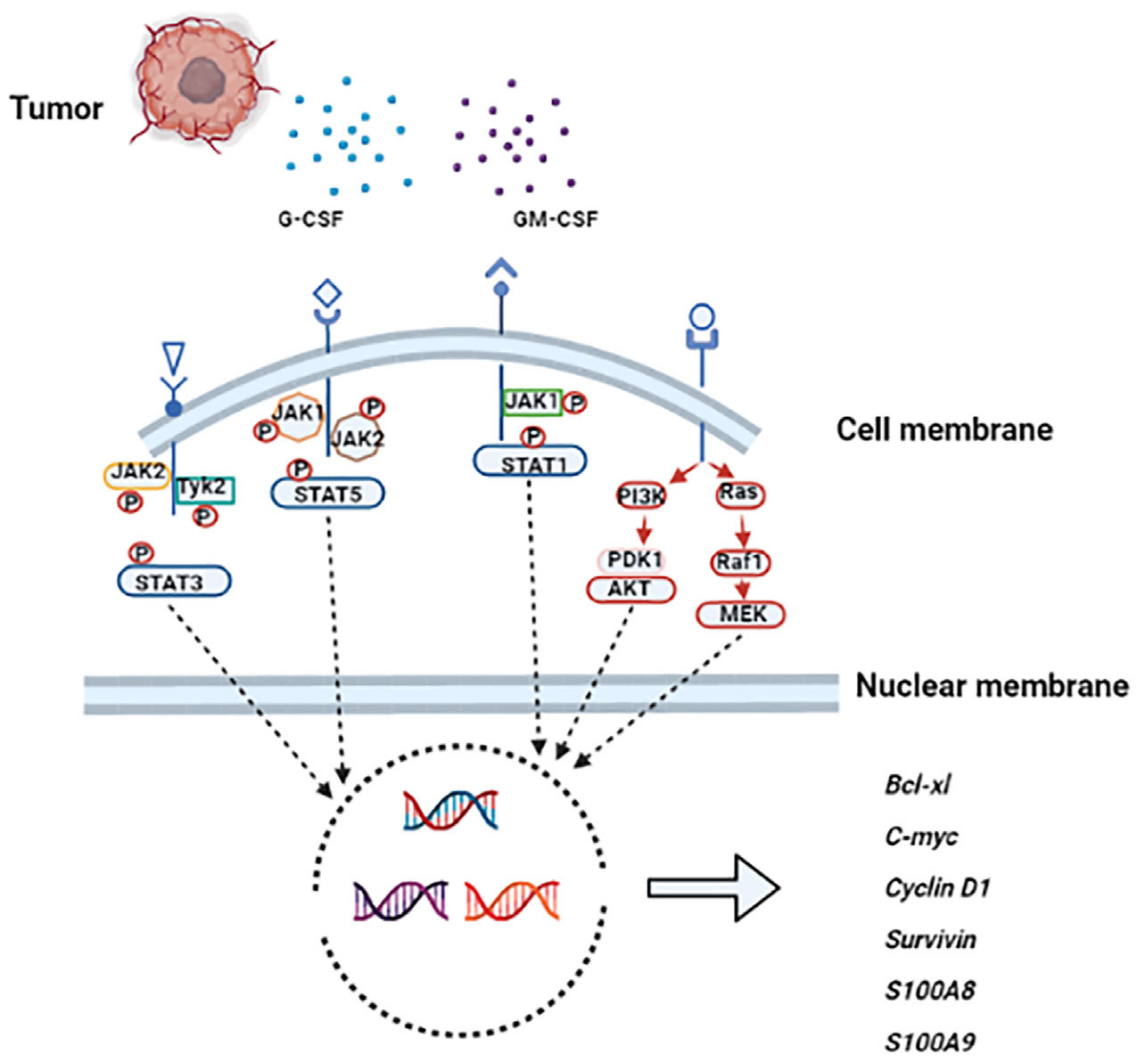
The mechanism of abnormal differentiation in hematopoietic stem cells driven by tumor‐derived G‐CSF and GM‐CSF. Granulocyte colony stimulating factor (G‐CSF) and granulocyte‐macrophage colony stimulating factor (GM‐CSF) have high affinity for their receptors, and then downstream signaling pathways are activated, including JAK/STAT, PI3K/AKT, and RAS/Raf1/MEK pathways. These signaling pathways play a significant role in regulating the differentiation and proliferation of hematopoietic stem cells (HSCs) by inducing the transcription of target genes such as *Bcl‐xl*, *C‐myc*, *Cyclin D1*, *Survivin*, *S100A8*, and *S100A9*. [Colour figure can be viewed at wileyonlinelibrary.com]

G‐CSF/GM‐CSF, two recognized cytokines involved in the regulation of hematopoiesis, can control the self‐renewal of HSCs and regulate the differentiation of HSCs into specific lineages. What’s more, G‐CSF and GM‐CSF were identified as “lineage‐specific” cytokines regulating progenitors in the granulocyte, granulocyte/macrophage lineages, respectively [56]. In general, G‐CSF/GM‐CSF mediated HSCs proliferation and mobilization requires specific interactions between the G‐CSF/GM‐CSF and granulocyte colony‐stimulating factor receptor/granulocyte‐macrophage colony‐stimulating factor receptor (G‐CSFR/GM‐CSFR). Important signaling molecules that mediate transduction of G‐CSFR responses include JAK1/2 kinases, Tyk2, the Src kinases p55^lyn^ and p56/59^hck^, STAT1, STAT3, and STAT5, as well as components of the p21^ras^/Raf/mitogen‐activated protein kinase pathway [57]. However, in a tumor‐bearing host, G‐CSF and GM‐CSF showed an opposite effect on the proliferation and mobilization of HSCs. G‐CSFR/GM‐CSFR belongs to the receptor tyrosine kinase signaling system. As shown in Fig. 2, in cancer patients the interaction between G‐CSF/GM‐CSF and G‐CSFR/GM‐CSFR could cause receptor dimerization, tyrosine phosphorylation, and the subsequent interaction with multiple intracellular signaling pathways such as Ras, MAPK, PI3K, JAK [58], finally inhibiting normal differentiation of HSCs and inducing the production of tumor‐promoting cell phenotypes [59].

### G‐CSF/GM‐CSF‐induced MDSCs proliferation and mobilization differentiation

MDSCs are characterized by their myeloid origin, immature state, and immunosuppressive ability, mainly including M‐MDSCs and PMN‐MDSCs [60]. They play key roles in the pathogenesis of cancers, chronic infection, autoimmune diseases, and transplantation. The amount of MDSCs is low in the circulation of healthy individuals and their role involves the regulation of immune responses and tissue repair [61]. In cancer patients, a tumor‐driven microenvironment characterized by changes in cytokine homeostasis arises [62], which blocks the differentiation from HSCs to mature cell types, replacing it with a large number of MDSCs. Various extracellular factors can induce MDSC differentiation such as macrophage colony‐stimulating factor (M‐CSF), G‐CSF, GM‐CSF, stem cell factor (SCF), and others. The administration of GM‐CSF in metastatic melanoma patients significantly increases CD14^+^HLA^−^DR^−/low^ MDSCs in the peripheral blood [54]. In addition, the administration of recombinant G‐CSF in mice causes the accumulation of Gr‐1^+^CD11b^+^ MDSCs and Tregs in the peripheral lymphoid organs [63]. In solid tumor, G‐CSF and GM‐CSF trigger activating pathways that involve JAKs and STATs. STAT3 is generally considered a key mediator regulating MDSCs expansion, as it promotes myelopoiesis and inhibits myeloid cell differentiation [60]. A novel study has indicated that GM‐CSF could promote the differentiation of suppressive monocytes, including M‐MDSCs, through two signaling pathways. First, a functional IFN‐γR1/2 signaling platform is formed on the cell surface that increases synthesis of IRF‐1 and allows its nuclear translocation. Second, activation of the PI3K pathway leading to phosphorylation of AKT, mTOR, S6, and 4E‐BP1 [64]. Moreover, interferon regulatory factor‐8 (IRF‐8) has been identified as an important ingredient of myeloid differentiation and lineage commitment. In breast cancer patients, the level of IRF‐8 declined with increasing MDSCs frequency, implying that it negatively regulates MDSCs [65]. Evidence has displayed that tumor‐derived G‐CSF and GM‐CSF accelerate IRF‐8 downregulation via STAT3‐ and STAT5‐dependent pathways [65].

### G‐CSF/GM‐CSF‐induced TANs proliferation and activation

Neutrophils are widely present in the human peripheral blood circulation. Especially when the host is injured or infected, G‐CSF will mobilize and expand neutrophils. Therefore, G‐CSF has been used to support patients receiving chemotherapy [66]. But clinical case reports have indicated that increased NLR in the peripheral blood of cancer patients and higher NLR is associated with more advanced or aggressive disease [67]. Accumulating evidence found that high‐density TANs are associated with poor prognosis of solid tumors [68]. Thus, TANs have been regarded as critical immunosuppressive cells in TME. Once TANs accumulate in tumor tissues, they show functional heterogeneity and the existence of two polarized states, namely, N1 and N2 [37], similar to monocyte polarization [69]. Notably, N2‐like TANs exhibit tumor‐promoting activity; however, N1‐like TANs show cytotoxicity to tumor cells, and a majority of TANs are characterized by the N2 phenotype.

Tumor‐derived G/GM‐CSF play a critical role in the activation and differentiation of TANs. G‐CSF could induce TANs to generate NETs, which has been confirmed to possess a dual effect on tumor growth [44]. Moreover, the expression of Bv8/prokinectin2 in tumor‐infiltrating neutrophils was upregulated, promoting tumor angiogenesis after being treated with G‐CSF [70]. OncostatinM (OSM) is a member of the IL‐6 family, which stimulates angiogenesis by enhancing FGF‐2 expression. GM‐CSF could induce TANs to secrete OSM in patients with breast cancer [67]. Hepatocellular carcinoma (HCC)‐derived GM‐CSF accelerates tumor progression through stimulating TANs to produce hepatocyte growth factor and activate hepatocyte growth factor/Met axis [71]. Interestingly, a study has revealed that in patients with lung cancer, IFN‐γ and GM‐CSF mediated APC‐like hybrid neutrophils generation by downregulating the ikaros transcription factor, and these APC‐like hybrid neutrophils could differentiate into protumor TANs [72].

### G‐CSF/GM‐CSF‐induced TAMs proliferation and activation

It has long been assumed that TAMs originate from monocytes/macrophages generated from HSCs; these cells are heterogeneous and versatile that could undergo their phenotypic/function dynamics in response to microenvironmental signals [73]. TAMs are the primary components of TME and act as a critical role in tumor metastasis, immunosuppression, and therapeutic resistance. TAMs are highly infiltrative and are not terminally differentiated, thus having the ability to polarize into distinct directions of M1 or M2 phenotype [74]. M1 macrophages secrete proinflammatory cytokines such as tumor necrosis factor‐α (TNF‐α), IL‐12, and IL‐6, which promote tumor cell killing by activating T‐cells [75, 76]. On the contrary, M2 macrophages produce several antiinflammatory cytokines such as IL‐10, IL‐13, IL‐4, and TGF‐β that promote tumor metastasis [77, 78]. A majority of TAMs are characterized by the M2 phenotype. Accumulating evidence suggests that the M1/M2 polarization state could be regulated by various endogenous cell signaling pathways, including C‐Jun N‐terminal kinase (JNK), PI3K/Akt, Notch, and JAK/STAT pathways [79]. GM‐CSF is a traditional hematopoietic stimulating factor and mainly produced by tumor and immune cells [80]. JAK/STAT signaling is an important oncogenes pathway in tumor cells [81, 82, 83, 84, 85, 86, 87, 88]. STAT5 has been linked to both M1 and M2 macrophage polarization in different models [89] and is activated in ∼31% of TAMs [90]. GM‐CSF is a well‐studied activator of STAT5 signaling [91, 92]. GM‐CSF‐induced cell differentiation and survival is via JAK2/STAT5‐Bcl‐2 and PI3K pathways [93]. *In vivo* and *in vitro* data confirmed that the activation of the GM‐CSF/STAT5 signaling pathway contributes to the progression of tumor and M2 phenotype polarization of TAMs [90]. In a mouse model of cancer, GM‐CSF has been described to induce an M1 phenotype by hyperactivating the RBP‐J/Notch pathway [79]. However, evidence also revealed that high expression of CD206, a marker of M2 macrophages, was a salient feature of GM‐CSF‐induced macrophages [94]. These results show that the role of GM‐CSF in TAMs polarization is complex, which may be related to the synergistic effect of other cytokines.

## Prognostic role of G‐CSF/GM‐CSF‐induced hematopoietic dysfunction in cancer metastasis

**Fig. 3:**
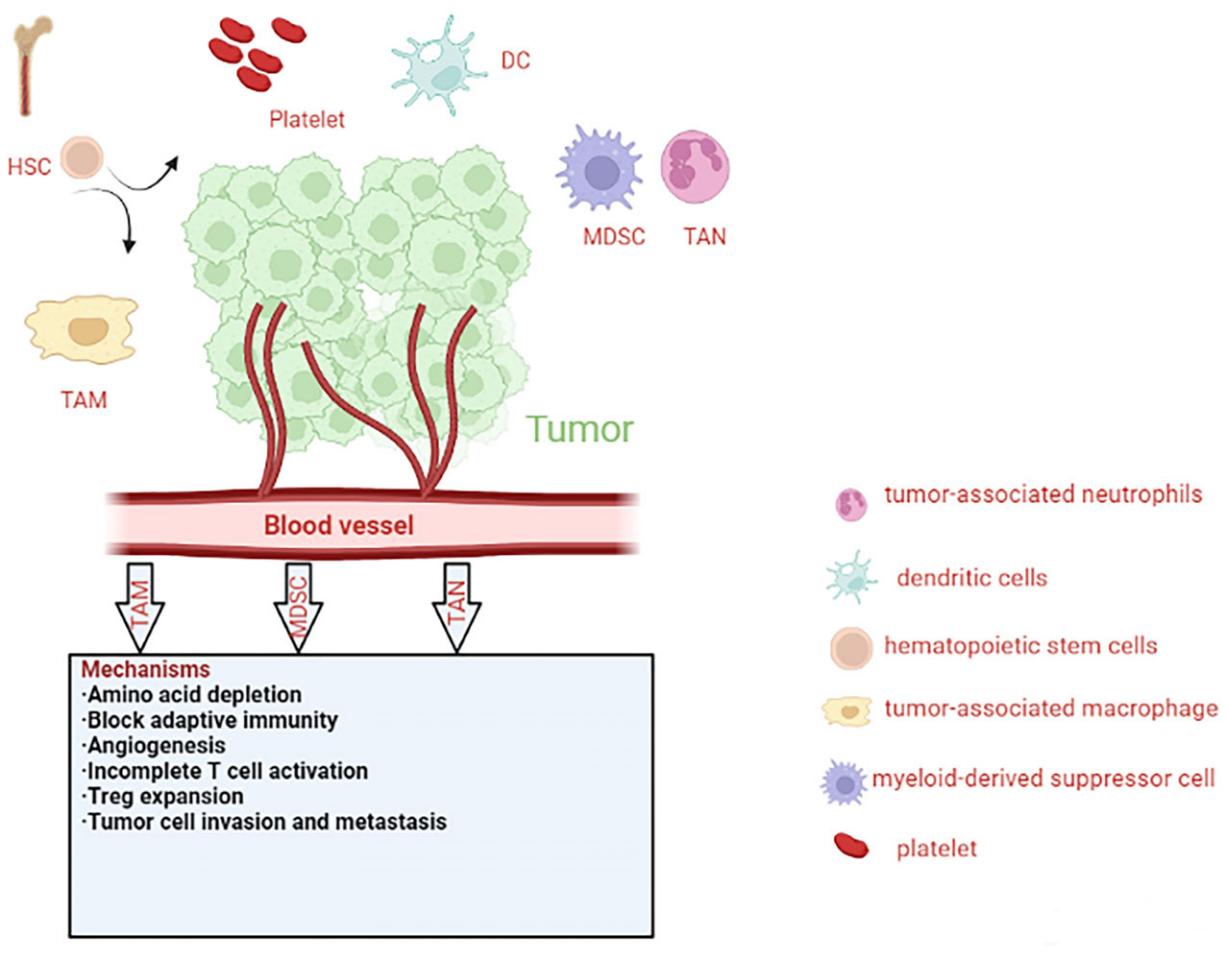
Tumor‐promoting effects and mechanisms of tumor‐associated cells. Tumor‐associated macrophages (TAMs), tumor‐associated neutrophils (TANs), and myeloid‐derived suppressor cells (MDSCs) are the main ingredients of the tumor microenvironment (TME). These cell types promote tumor invasion and metastasis through angiogenesis, regulatory T‐cell expansion, and incomplete T‐cell activation. HSC, hematopoietic stem cell; DC, dendritic cell; Treg, regulatory T‐cell. [Colour figure can be viewed at wileyonlinelibrary.com]

Normally, the development of blood cell lineages is tightly controlled by endogenous signals that drive the differentiation of HSCs to highly proliferative lineage‐committed progenitors [95, 96, 97, 98]. These myeloid progenitors can differentiate into iMCs, the latter migrate to peripheral organs where they differentiate into macrophages, dendritic cells (DCs), or granulocytes. However, recent studies have indicated that tumors are closely related to a profound perturbation in myelopoiesis. Tumor‐derived factors could regulate the differentiation of HSCs and subsequently cause hematopoietic dysfunction [10]. As shown in Fig. 3, among the cytokines secreted by tumors, GM‐CSF and G‐CSF could not only promote the myeloid‐biased differentiation, but also induce the differentiation of myeloid precursors into functional TAMs, TANs, or MDSCs [99, 100], and these cell types could negatively regulate immune responses and facilitate tumor metastasis and angiogenesis [60, 101]. These findings are consistent with clinical studies, revealing that the concentrations of these factors are upregulated in patients with tumor, and this level is highly correlated with poor prognosis in various tumors, as summarized in Table 3 [102, 103].

**Table 3:** Roles of G‐CSF and GM‐CSF in solid cancers. G‐CSF, granulocyte colony stimulating factor; GM‐CSF, granulocyte/macrophage colony stimulating factor; MDSC, myeloid‐derived suppressor cells.

Tumor type	G‐CSF	GM‐CSF
Prostate cancer	Increase cancer stem cell phenotype	
Melanoma	Tumorigenic	Antiangiogenic or induction of MDSCs
Colorectal cancer	Tumorigenic	immune‐independent mediated antitumor effect
Bladder carcinoma	Autocrine growth	
Glioma	Angiogenic, induction of MDSCs, autocrine/paracrine growth	
Lung cancer	Angiogenic, induction of MDSCs	
Hepatocellular carcinoma	Accumulation of MDSCs, splenic EMH	Accumulation of MDSCs, splenic EMH
Acute myeloid leukemia	Proliferation of leukemia cells or provide better chemotherapy response	Proliferation of leukemia cells or neutrophils recovery

Lung cancer is the most common malignant cancer driven by ectopic secretion of G‐CSF/GM‐CSF, including primary and metastatic types [104, 105, 106, 107]. Increased G‐CSF levels are considered to be a sign of shortened survival in non‐small‐cell lung cancer (NSCLC) patients [106, 108]. Interestingly, microarray data indicated that the expression of the GM‐CSF gene was increased in small‐cell lung cancer but not in NSCLC, implying the posttranscriptional mechanism of cytokine accumulation [109]. Various cell types have been considered to be a source of G‐CSF/GM‐CSF in lung cancer. In particular, tumor‐associated endothelial cells and Gr‐1^+^CD11b^+^ MDSCs are mainly responsible for the secretion of these cytokines [110, 111]. A clinical study suggested that NK cells decreased, while the accumulation of MDSCs increased in patients with lung cancers [112]. There was evidence that PMN‐MDSCs have emerged as an independent prognostic factor for survival in NSCLC, inhibiting the activation of CD8^+^ T lymphocytes by producing high levels of Arg‐1 and inducible iNOS [113, 114]. However, other clinical research indicates that the increased percentage of new M‐MDSCs characterized by CD14^+^ CD15^+^ CD11b^+^ CD33^+^ HLA‐DR^−^ Lin^−^ was correlated with poor prognosis in patients with NSCLC [115]. Furthermore, monocyte and neutrophil counts were higher in peripheral blood in lung cancer patients, especially when these patients had histories of smoking, drinking, and liver metastasis [112]. Smoking NSCLC patients showed a high proportion of M2 macrophages that induced rejection of cisplatin treatment through activation of JAK1/STAT1/NF‐κB/Notch‐1 and ERK1/2/FRA‐1/slug signaling pathways [116, 117, 118]. It is likely that specific subsets of neutrophils, TANs, are associated with inactivated CD8+ T‐cells, leading to poor survival [119]. Given that G‐CSF/GM‐CSF may promote the progression and distant metastasis of lung cancer, using these cytokines as adjuvant therapy should be carefully considered.

Glioma is the most frequently occurring type of malignant brain tumor, including glioblastoma (GBM), the highest‐grade primary central nervous system cancer [120]. An increased ratio of NLR is a common phenomenon in GBM. GBM cells synthesize GM/G‐CSF stimulate bone marrow to shift hematopoiesis toward granulocytic lineages and away from lymphocytic lineages; this shift is immunosuppressive [121]. Increased G‐CSF(R)/GM‐CSF(R) levels have been confirmed to correlate with a higher tumor grade [122, 123]. In these tumors, G‐CSF(R)/GM‐CSF(R) promote progression mainly using auto‐/paracrine activation of proangiogenic pathways by activating STAT3 or increasing the expression of VEGF/VEGFR [124, 125, 126, 127]. In the GBM model, decreased G‐CSF/GM‐CSF levels inhibit cancer cell invasion and proliferation, thereby implying the regulatory function of these cytokines on TME [128]. In addition, it has been shown that the accumulation of MDSCs is related to the increase of G‐CSF levels in glioma patients [129]. Patients with GBM have increased MDSC counts (CD33^+^HLA‐DR^−^) in their blood that are composed of neutrophilic (CD15^+^; >60%), lineage‐negative (CD15^−^CD14^−^; 31%), and monocytic (CD14^+^; 6%) subsets. And these MDSCs promote the progression of GBM by suppressing T‐cell IFN‐γ generation [129]. A study indicated that the frequency of M2 macrophage/microglia was increased in mesenchymal GBM and is associated with mesenchymal glioma cell differentiation, which induced a poor response to ionizing radiotherapy [130].

G‐CSF was initially extracted from the human bladder carcinoma cell line 5637, suggesting an important role in progression of bladder malignancies [131]. An autocrine growth occurred after G‐CSFR had been integrated into the G‐CSF‐secreting tumor cells from a resected bladder carcinoma [132]. Previous studies have revealed that bladder tumors that secrete G‐CSF/GM‐CSF or express their specific receptors are uncommon and have significant differences in response to treatment [133, 134, 135, 136]. Microarray data report that compared with normal tissues, the gene expression of GM‐CSF and GM‐CSFRα in bladder tumors has a statistically significant increase; on the contrary, the changes of G‐CSF and G‐CSFR have not yet been found [137, 138]. A clinical study indicated that peripheral blood mononuclear cell (PBMC) from bladder cancer patients contain different myeloid suppressor cell subsets, which produced various proinflammatory chemokines/cytokines including CCL2, CCL3, CCL4, IL‐8, and IL‐6, or inhibited T‐cells proliferation by induction of CD4^+^ Foxp3^+^ Tregs [139]. Furthermore, an *in vitro* study suggested that M2 macrophages could promote human urothelial bladder cancer (UBC) T24 cells survival [140]. These results showed that inflammation and immune dysfunction contribute to the progression of bladder cancer.

Various studies suggest an increased frequency of M2 macrophages in colorectal cancer (CRC) patients, which then induced the progression and liver metastasis of CRC through promoting tissue remodeling, angiogenesis, and immune dysfunction [141, 142]. Within the TME, neutrophils polarized towards N2 subsets, which aggravated the invasion and metastasis of CRC by the production of MMP‐9, VEGF, HGFs, and NETs [143]. Accumulating evidence has demonstrated that MDSCs in the peripheral blood and tumor tissues are associated with tumor stage, histological grade of cancer, and lymph node metastases in CRC [144]. MDSCs aggravated CRC resistance to immunotherapy via producing high levels of the immune mediators Arg‐1, iNOS, and Nox2, associated with maturation and activation of T‐cells [145]. Elevated GM‐CSF in plasma was found in patients with CRC; unlike in other malignant tumors, ectopic secretion of GM‐CSF driven by demethylation of its gene promoter has demonstrated an immune‐independent mediated antitumor effect [146]. In addition, the 5‐year overall survival rate of CRC patients whose tumors tested positive for GM‐CSF/GM‐CSFR has improved [146]. In contrast, patients with colon and rectal cancers accompanied by G‐CSF‐secretion have shown a high ratio of large tumors and distal metastases, and the overall survival of these patients is poor, suggesting oncogenic effects for G‐CSF [147, 148].

Melanoma is a highly malignant cancer with a tendency to metastasize early. A clinical case study reports the presence of severe G‐CSF secretory melanoma [149]. *In vitro*, although melanoma cells were found to express the G‐CSFR transcript, the enhancement of cell proliferation and invasion do not occur on G‐CSF stimulation, suggesting an absence of the G‐CSFR protein [150]. However, the role of GM‐CSF in melanoma is controversial. In a murine melanoma model, TAMs secreted a soluble VEGF receptor to inactivate VEGF and inhibit angiogenesis after stimulation with GM‐CSF under hypoxic conditions [151]. Congruently, adjuvant GM‐CSF monotherapy in advanced melanoma patients revealed a reduction in the melanoma‐specific deaths [152]. But other studies also found that there exist positive correlations between GM‐CSF and tumor progression; for example, GM‐CSF can exert tumorigenic effects via mediating MDSCs infiltration in a transgenic mouse melanoma model [153]. Multiple reports have highlighted MDSCs as immunotherapy inhibitors of melanoma [154]. One study has found that decreased expression of T‐cell receptor (TCR) ζ‐chain was a major feature of T‐cell dysfunction caused by MDSCs in a mouse model of melanoma [153]. Moreover, MDSCs could induce macrophage reprogramming by suppressing CD40/IL27 signaling, thereby promoting melanoma progression [155]. TAMs maintain an immunosuppressive M2 subset through the PD‐1/PD‐L1 pathway; these M2 macrophages promote melanoma progression by recruiting immunosuppressive cells and inhibiting T‐cell activation [156]. There was evidence that high levels of M2 macrophages were associated with a poor prognosis of melanoma [157, 158]. Evidence suggests that extracellular vesicles (EVs) derived from metastatic human melanoma cell line (MV3) induce neutrophil chemotaxis, promoting formation of NETs and ROS, driving these cells to a protumor/N2 polarization through the CXCR2/PI3K‐Akt axis [159].

G‐/GM‐CSF can enhance the dissemination and bone metastasis in prostate cancer. G‐CSF increases a cancer stem cell phenotype through upregulation of octamer‐binding transcription factor 3/4 (Oct3/4), nanog homeobox pseudogene 8 (NANOGP8), and ATP‐binding cassette transporter G2 (ABCG2) [160]. GM‐CSF facilitates bone metastasis via increasing osteoclastic activity [161]. Emerging evidence also reveals that Tregs inhibit the function of antigen‐presenting cells by interacting with CD80/CD86 via their surface cytotoxic T lymphocyte‐associated antigen 4 (CTLA‐4) receptor, and secrete granulase B perforating protein, which suppresses the antitumor function of T‐cells [162]. The underlying mechanisms of TAMs in the regulation of prostate cancer initiation and progression are complex. Some studies have suggested that in prostate cancer, TAMs mediated angiogenesis and bone metastasis by activating CD204/NF‐κB and the MFG‐E8 pathway, respectively [163, 164]. The role of MDSCs in prostate cancer is still unclear. Currently, activation of the CD40/CD40L and PI3K/PTEN/Akt pathway, and promotion of M2 macrophages polarization are considered to be highly correlated with the protumor function of MDSCs [165].

Hepatocellular carcinoma (HCC) is a primary liver malignancy with a high global prevalence and a dismal prognosis. Immune system instability is a major clinical performance of HCC [166]. Bone marrow hematopoiesis and EMH are the two major ways for maintaining immune system homeostasis. Notably, EMH has also been recognized in benign or malignant hepatic tumors, such as hepatoblastoma, hepatocellular adenoma, HCC, and vascular tumors [167]. The spleen is an important site for EMH and tumor immunotolerance. There was evidence that in a murine H22 subcutaneous hepatoma model, spleen weight was identified to be positively correlated with tumor weight, and the proportion of CD8^+^ T lymphocyte in the spleen were decreased, while the percentages of M‐MDSCs, CD11b^+^ F4/80^+^ macrophages and PMN‐MDSCs at day 21 after tumor cell inoculation [168]. Moreover, a study indicated that icaritin could induce antitumor immune responses in HCC by inhibiting splenic MDSCs generation [169]. Recent studies revealed that tumor‐derived factors, including GM‐CSF and G‐CSF, mediate systemic deviation of hematopoiesis in extramedullary tissues such as the spleen [2]. GM‐CSF and G‐CSF have been described to cause an accumulation of MDSCs in the spleen of HCC mice, and an increased serum GM‐CSF level has been described in patients with HCC [170]. Furthermore, a study revealed that chemerin has a protective role in HCC by inhibiting the expression of IL‐6 and GM‐CSF and MDSC accumulation [171]. These results suggest that GM‐CSF/G‐CSF mediate splenic EMH and MDSCs accumulation and play a critical role in the progression and immunosuppression microenvironment of HCC.

AML is a complex, heterogeneous hematological malignancy caused by mutations in immature myeloid cells differentiation and proliferation [172]. EMH in the spleen is a characteristic feature of the chronic myeloproliferative disorders (CMPDs) and various other neoplastic or reactive myeloid conditions [173]. Both AML and CMPDs have a variety of underlying cytogenetic defects; allelic losses were common in the neoplastic EMH found in the spleens of CMPDs and AML [174]. A study showed that in an AML mouse model with high cyclin A1 levels, a majority of immature myeloid cells infiltrated the spleen and liver [175]. It is also of interest that the microenvironment of spleen in an AML mouse model has a promoting effect on the activation and accumulation of NK cells, although the underlying mechanism is unclear [176]. A meta‐analysis revealed that treatment with chemotherapy plus G‐CSF appears to provide better survival and treatment responses, particularly for patients with previously untreated AML [177]. Most clinical trials have indicated that GM‐CSF accelerated neutrophils recovery and reduced early mortality in high‐risk patients with AML [178, 179]. However, the abnormal signal transduction caused by GM‐CSF/G‐CSF in AML patients was frequently reported; leukemic cells have surface receptors for CSFs and proliferate in response to CSFs [180]. These results suggest that a phenomenon of splenic EMH does exist in AML, and the effect of GM‐CSF/G‐CSF on AML is dual directional. There is currently no evidence that this dual function is involved in splenic EMH.

## Conclusion

Tumor‐associated hematopoietic disorders work through a feedback mechanism within the TME and participate in the formation of the tumor immunosuppressive microenvironment. Based on a series of data highlighting the interplay of tumor growth, angiogenesis, and hematopoietic dysregulation, it is generally believed that abnormal hematopoiesis can accelerate tumor progression by inducing the generation and activation of tumor‐associated immunosuppressive cells. G‐CSF/GM‐CSF‐induced hematopoietic dysregulation can be used as a marker of poor prognosis in various tumors. However, it should be noted that many different local, systemic mechanisms and regulatory pathways have been implicated in hematopoietic dysregulation during tumor evolution, and the exact mechanism of this regulation is still not fully understood. Therefore, improving dysregulated hematopoietic environments based on the elimination of tumor‐induced abnormal immunosuppressive cells may be a valuable therapeutic approach.

## Conflict of interest

The authors declare no conflicts of interest.

## Author contributions

J‐LG and G‐Y Lv designed and revised the review, K He, R Hoffman, X Liu and R‐Z Shi wrote the manuscript.) and G‐YL (lv.gy@263.net) designed the project. KH, RDH, XL, and R‐ZS wrote the article.
